# Dominance of non-wetland-dependent pollinators in a plant community in a small natural wetland in Shimane, Japan

**DOI:** 10.1007/s10265-023-01518-9

**Published:** 2024-01-11

**Authors:** Tomohiro Watazu, Masayoshi K. Hiraiwa, Masahito Inoue, Hideo Mishima, Atushi Ushimaru, Tetsuro Hosaka

**Affiliations:** 1 Development Technology Course, Graduate School for International Development and Cooperation, Hiroshima University, 1-5-1 Kagamiyama, Higashi-Hiroshima City, Hiroshima 739-8529 Japan; 2 Department of Environmental Management, Faculty of Agriculture, Kindai University, 3327-204 Nakamachi, Nara, 631-8505 Japan; 3 The Shimane Nature Museum of Mt. Sanbe, 1121-8 Tane, Sanbe-cho, Ohda, Shimane 694-0003 Japan; 4 Graduate School of Human Development and Environment, Kobe University, 3-11 Tsurukabuto, Nada, Kobe, 657- 8501 Japan; 5 Transdisciplinary Science and Engineering Program, Graduate School of Advanced Science and Engineering, Hiroshima University, 1-5-1 Kagamiyama, Higashi-Hiroshima, 7398529 Japan

**Keywords:** Bumblebee, Diptera, Hover fly, Muscoid fly, Pollination syndrome, Pollinator community

## Abstract

Many wetland plants rely on insects for pollination. However, studies examining pollinator communities in wetlands remain limited. Some studies conducted in large wetlands (> 10 ha) have suggested that wetland-dependent flies, which spend their larval stage in aquatic and semi-aquatic habitats, dominate as pollinators. However, smaller wetlands surrounded by secondary forests are more prevalent in Japan, in which pollinators from the surrounding environment might be important. Additionally, information regarding floral traits that attract specific pollinator groups in wetland communities is scarce. Therefore, this study aimed to understand the characteristics of insect pollinators in a small natural wetland (2.5 ha) in Japan. We examined the major pollinator groups visiting 34 plant species and explored the relationship between the flower visitation frequency of each pollinator group and floral traits. Overall, flies were the most dominant pollinators (42%), followed by bees and wasps (33%). Cluster analysis indicated that fly-dominated plants were the most abundant among 14 of the 34 target plant species. However, 85% of the hoverflies, the most abundant flies, and 82% of the bees were non-wetland-dependent species, suggesting that these terrestrial species likely originated from the surrounding environment. Therefore, pollinators from the surrounding environment would be important in small natural wetlands. Flies tend to visit open and white/yellow flowers, whereas bees tended to visit tube-shaped flowers, as in forest and grassland ecosystems. The dominance of flies in small wetlands would be due to the dominance of flowers preferred by flies (e.g., yellow/white flowers) rather than because of their larval habitats.

**Supplementary Information:**

The online version contains supplementary material available at 10.1007/s10265-023-01518-9.

## Introduction

Many wetland plants have become endangered worldwide due to declining and shrinking of natural wetlands (Ramsar Convention Secretariat 2018; Shimoda and Nakamoto 2003). Conservation of remaining population of those wetland plants is an urgent issue, and understanding of their reproduction systems is essential. Insects play a vital role in pollinating many wetland plants with sexual reproduction (Kato 1998; Kato and Miura 1996). While asexual reproduction such as vegetative reproduction can efficiently increase clonal individuals (Da Silva et al. 2017; Holsinger 2000), sexual reproduction is very important for maintaining their genetic diversity (Feigs et al. 2022; Jeltsch et al. 2013). Genetic diversity plays an important role in the survival of species because it gives species the ability to adapt in a changing environment (Ouborg et al. 2006). Therefore, understanding the relationships between plants and pollinator insects is essential for conservation of wetland plant communities (Kato 1998; Kato and Miura 1996).

Studies on wetland pollinators have often been focusing on bees and wasps (e.g. Bartholomew and Prowell 2006; Hall and Ascher 2010; Moroń et al. 2008; Pascarella et al. 2000; Pindar and Raine 2023; Purvis et al. 2020; Stephenson et al. 2018; Vickruck et al. 2019), and studies covering pollinator communities of multiple taxa are still limited (but Kato and Miura 1996; Mahoro et al. 2008; Negoro 2004; Nkurikiyimana et al. 2023). However, the previous studies on pollinator communities indicate that flies often dominate as pollinators in wetlands (Kato and Miura 1996; Mahoro et al. 2008), unlike in forests and grasslands where bees are more prevalent (Shinjo et al. 2014). Specifically, wetland-dependent hover flies, which spend their larval stage in aquatic and semi-aquatic habitats, were dominant among flies (Kato and Miura 1996; Mahoro et al. 2008). Among bees, wetland-dependent bees such as *Ceratina* bees (Anthophoridae), which nest on reed stems, were abundant (Kato and Miura 1996). This suggests that wetland pollinators might be dominated by wetland-dependent flies and bees, of which larva requires wetland environments.

However, it is worth noting that some wetland plants are pollinated by non-wetland dependent halictid bees and bumblebees that nest on terrestrial lands, possibly in nearby forests or grasslands (Kato and Miura 1996; Mahoro et al. 2008). Although the studies conducted so far have mainly focused on relatively large wetlands (ca. 16–25 ha), Japan is home to a considerably greater number of small wetlands surrounded by natural or planted forests (Fukui et al. 2012; Koike et al. 2003). In these smaller wetlands, pollinators from the surrounding environment may be more abundant than in larger wetlands. If this is indeed the case, it is important to consider the surrounding environment as a conservation target in wetland conservation efforts. Taki et al. (2011) reported that the abundance of pollinator bees in crop fields was higher when the surrounding area was natural forest than plantation. Identifying key pollinators for plant communities in wetlands and their associated habitats (both wetland and non-wetland-dependent) is essential for developing effective conservation strategies for wetland plant communities (Begosh et al. 2020; Kato and Miura 1996; Mahoro et al. 2008). However, data on pollinator communities in small natural wetlands are still limited.

Many studies have investigated the relationship between floral traits and pollinators in forests and grasslands (Gong and Huang 2011; Hegland and Totland 2005; Ishii et al. 2019; Lázaro et al. 2008; Nakano and Washitani 2003; Ollerton et al. 2009; Pickering and Stock 2008; Reverté et al. 2016), but only a few studies have included wetlands (e.g. Olesen and Jordane 2002; Subedi et al. 2021). It is generally accepted that plants possess diverse floral traits, such as morphology, size, bloom volume, color, nectar guide, and scent, and rewards such as pollen, nectar, and flower oil to attract specific pollinators (Feagri and van Pijl 1971). Flowers visited by the same group of pollinators often exhibit common floral traits that are shared across plant taxonomic groups, which is often referred to as pollination syndromes (Faegri and van der Pijl 1971; van der Pijl 1960; Wyatt 1983). Pollination syndromes have been studied mainly in forests and grasslands. For instance, it has been observed that flies often visit open-shaped white/yellow flowers (An et al. 2018; Gong and Huang 2011; Hegland and Totland 2005; Lázaro et al. 2008; Luana 2014; McCall and Primack 1992; Pickering and Stock 2008; Shrestha et al. 2016), whereas bees prefer tube-shaped flowers of various colors, with bumblebees preferring blue flowers (Gong and Huang 2011; Hegland and Totland 2005; Lázaro et al. 2008; Westerkamp and Bockhoff 2007). However, these relationships are highly influenced by the local plant and pollinator community structure (Hingston and McQuillan 2000; Ishii et al. 2019; Lazzaro et al. 2008; McCall and Primack 1992; Ollerton et al. 2009). Consequently, if wetlands harbor unique pollinator compositions (e.g., dominance of flies), they may exhibit distinct pollination syndromes compared to other ecosystems. Investigating whether the pollination syndromes of wetland plants follow the general patterns would yield valuable insights into plant-pollinator interactions within wetland communities.

Therefore, in this study, we aimed to quantify the visitation frequency of pollinators for 38 plant species in Akana Wetland, a small natural wetland surrounded by secondary forest in Shimane Prefecture, Japan. Our goal was to understand plant-pollinator relationships at the community level in the wetland by examining the pollinator composition of each plant species and pollinator preferences for floral traits. Specifically, we sought to answer the following questions:

(1) Do flies dominate the pollinator community in this small natural wetland?

We predicted yes, but bees might abundant as well due to the small size of the wetland.

(2) Are non-wetland-dependent bees and flies more abundant than wetland-dependent ones?

We predicted yes, because many pollinators would come from outside in the small wetland.

(3) How does the visitation frequency of each pollinator group relate to specific floral traits?

We predict that there is a relationship between pollinator groups and flower traits like in forest and grassland ecosystems.

## Materials and methods

### Study community

The survey was conducted in Akana Wetland, situated in Iinan Town, Shimane Prefecture (35°00′49″–00′51″N, 132°42′10″–42′22″E, 440 m a.s.l). The region is classified as a warm temperate region despite experiencing a considerable amount of snowfall in winter, with the average deepest snowfall being approximately 80 cm over the past 10 years. Akana Wetland, a natural conservation area in Shimane Prefecture, covers an area of approximately 2.5 ha, and is located in a valley within a gently sloping mountain range. The bedrock in this area is granodiorite, overlaid by ejecta from the Sanbe volcano, dating back 30,000 years. The accumulation of this ejecta created an aquifer where surface water transforms into groundwater, giving rise to the formation of wetlands (Inoue et al. 2009).

The vegetation surrounding Akana wetland predominantly consists of a *Rhododendron reticulatum–Pinetum densiflorae* community, which includes wetland plant species such as *Pogonia japonica* (Orchidaceae), *Platanthera tipuloides* (Orchidaceae), *Menyanthes trifoliata* (Menyanthaceae), and *Alnus japonica* (Betulaceae). Although minor human interventions such as mowing, maintenance of wooded paths, and minor repair work at the wetland’s edges have been conducted, no significant human disturbances have been recorded. The presence of *Menyanthes trifoliata*, a relict glacial plant found in wetlands (Hewett 1964), indicates that Akana Wetland has maintained its environmental conditions for a very long time.

### Data collection

We conducted a pollinator survey of 38 plant species that were dominant within and around the wetland from April to November 2019. The plant nomenclature used was in accordance with the YList (http://ylist.info/). We established 1 to 26 square plots (1 × 3 m) for each plant species depending on the area of flowering, to obtain data on flower density and flower visitation frequency of pollinators. We captured all pollinators visiting the flowers of the target plants during a 15-min period within each square plot. In this study, we defined pollinators as insects having direct physical contact with the stigmas and/or anthers. We conducted the survey on sunny or cloudy (rainless) days between 0830 and 1830 h. Each plant species was surveyed at least once in both the morning and afternoon. In total, we conducted the survey 258 times, amounting to a total of 3,870 min of observation.

We recorded data on various flower traits, including flower density, size, shape, color, and habitat type (Table S1). Flower density was quantified by counting the number of flowers on each plant in the square plot during the pollinator survey. For flower size, we haphazardly selected and measured the size of 10–25 flowers for each species. The area of attraction-related parts for circular flowers was calculated using πr^2^ (r = radius), whereas for square flowers it was determined using lw (l = length and w = width). For triangular flowers, we used lw/2, and for conical flowers, we used either πdr (d = radius of fan shape with cone expanded), πdr + πr^2^, πdr + lw, or πdr + lw/2 depending on their shape (Table S1). For Asteraceae species, such as *Cirsium sieboldii*, *Aster glehnii*, *A. yomena*, *Senecio pierotii*, and *Ixeridium dentatum*, each flower head was considered a single flower. For *Viburnum plicatum*, we included the area of the calyx, which is the decorative part of the flower, when determining the area of the flower. We also recorded the shape (whether they were tube-shaped or open) and the human-perceived color (blue-violet, white/yellow) for each species, following the methodology outlined by Hegland and Totland (2005). Our focal plants grew either in wetlands or at the boundary between wetlands and forests. The habitat type of each plant species was classified as either wetland or forest edge, according to the classification system developed by Miyawaki (1978).

We defined the flower visitation frequency of each pollinator as the number of individuals captured per plot per 15 min with reference to Hegland and Totland (2005). In addition, as a reference, we show the flower visitation frequency of each pollinator per 100cm^2^ flower area per 15 min for each plant (Table S2). The reason for calculating frequency of pollinators per flower area instead of per flower is that the visitation frequency of pollinators could be underestimated for species having inflorescences with many tiny flowers such as Apiaceae. The frequency of pollinators per flower area was also used by Hiraiwa and Ushimaru (2017). All captured pollinators were identified and categorized into the order groups except Hymenoptera, for which we separately classified bees and wasps, following Shinjo et al. (2014). To quantify the flower visitation frequency of the lower taxonomic groups of Hymenoptera and Diptera, we further classified Hymenopterans into bumblebees (Apidae: *Bombus*), introduced honey bees (*Apis mellifera*), other bees (Apidae: *Ceratina, Xylocopa*, *Amegilla*; Halictidae: *Lasioglossum*; *Halictus*; Megachilidae: *Megachile*), and wasps (Ichneumonidae: *Iseropus*; Vespidae: *Vespa*; Braconidae: *Urosigalphus*; Scoliidae: *Megacampsomeris*), following Lázaro et al. (2008). Dipterans were further classified into muscoid flies (Muscidae; Calliphoridae: *Stomorhina*; Tachinidae: *Siphona*, *Prosena*), hover flies (Syrphidae: *Episyrphus*, *Sphaerophoria*, *Paragus*), beeflies (Bombyliidae: *Systropus*, *Bombylus*), and other flies (Milichiidae; Tipulidae; Mycetophilidae), following Lázaro et al. (2008). We also identified the Dipterans and Hymenopterans at the family and genus levels, respectively, and further classified them into wetland and non-wetland-dependent species based on their larval habitat and nest site, with reference to Kato and Miura (1996).

### Data analysis

#### Major pollinators and their relationship with various flower traits

For all analysis, we used 34 (980 samples) out of the 38 plant species surveyed, from which we captured at least 10 individuals of pollinator insects per plant species. To identify the major pollinators and explore their relationship with various flower traits, we conducted hierarchical cluster analysis using Ward’s method to divide them into four groups at a height of 100 (Fig. S1). Using the Shannon-Wiener diversity index (*H’*), we further divided the bottom group in Fig. S1 into Diptera-Apoidea-mixed type (*H’* < 1.5) and generalist type (*H’* ≥ 1.5) (Table S2). From these, we classified the plant species into five entomophily types: Diptera-dominated type (fly-type), Apoidea-dominated type (bee-type), Diptera-Apoidea-mixed type (fly/bee-type), Coleoptera-dominated type, and generalist type (both Diptera and other insects were abundant).

To determine if there are differences in flower shape and color among entomophily types, we used Fisher’s exact probability test to examine the differences in the number of plant species having each flower shape (tube-shaped or open) and color (blue-violet, yellow/white) among entomophily types.

#### Relationship between the flower visitation frequency of flies and bees and various flower traits

We used generalized linear mixed models (GLMMs) using a Poisson error distribution and logarithmic link to investigate the relationship between the frequency of flower visitation by flies and bees and various flower traits. The response variable was the flower visitation frequency (the number of individuals captured per plot per 15 min) of flies or bees. The fixed variables included flower traits, such as flower density, size (average for each plant species), shape, color, habitat type, season (spring: April to June, summer: July to September, fall: October to November), and time of day (morning: 0830 to 0930 h, daytime: 0930 to 1600 h, evening: 1600 to 1830 h). To account for the phylogenetic relationships of plants, we included species, genus, and family identity as nested random variables in the GLMMs. We conducted AIC-based model selection starting from the full model. As a result, the full model was the best model for flies while the model excluding season and time of day was the best for bees (Table S3). We report the results based on these best models in the following section.

In addition, we examined the relationship between flower visitation frequency and floral traits for specific sub-groups (bumblebees and other bees, muscoid and hover flies), butterflies and coleopterans, provided that more than 80 individuals were captured for each group. Owing to the small sample size, GLMMs could not be applied, therefore, we plotted each pollinator group in two-dimensional coordinates using non-metric multi-dimensional scaling (NMDS) with the Morishita-Horn similarity index based on the number of pollinator individuals captured for each plant species. We visually assessed the relationship with flower size, density, shape, and color using a vector fitting analysis.

All statistical analyses were performed using R version 4.02 (R Core Team 2020). We used the *lme4* package (Bates et al. 2015) for GLMMs and the *vegan* package (Oksanen et al. 2022) for NMDS.

## Results

### Flower visitation frequency of pollinators

In total, we collected 983 pollinator insects (Table S4). The mean frequency of flower visitation by all pollinators ranged from 0 to 30.0 individuals per plot per 15 min (Table S2). Hymenoptera showed a range of 0 to 7.3 individuals, and Diptera showed a range of 0 to 4.0 individuals. The plant species with the highest total visitation frequency was *Viburnum dilatatum* (30.0 individuals), which was visited by several Coleopterans. *Frangula crenata* had a high visitation frequency of Hymenoptera (7.3 individuals), whereas *Cicuta virosa* had a high frequency of Diptera (4.0 individuals).

Regarding the flower visitation frequency of each pollinator per 100cm^2^ flower area per 15 min for each plant, the plant species with the highest total visitation frequency was *Frangula crenata* (Table S2). *Frangula crenata* also had a high visitation frequency of Hymenoptera, whereas *Sagittaria aginashi* had a high frequency of Diptera. In contrast, no pollinators were observed on the flowers of *Platanthera tipuloides* (Orchidaceae), *Utricularia bifida* (Lentibulariaceae), and *U. uliginosa* (Lentibulariaceae) during observations that lasted from 60 to 105-min for each plant species.

### Pollinator community

The percentage distribution of each pollinator order among the total number of pollinator individuals was as follows: Diptera 42%, Hymenoptera 33%, Coleoptera 16%, Lepidoptera 8%, and other orders 1% (Table S4). Among the sub-taxonomic groups, hover flies were the most common, accounting for 27% of all pollinators (Table S4). Furthermore, 85% of the hover flies observed were non-wetland-dependent, meaning they spend their larval stage on land (Table S5). Similarly, 82% of the bees observed were non-wetland-dependent bumblebees, which nest in the ground (Table S6). Non-wetland-dependent pollinators were dominant in all plant species except *Viola verecunda* var. *verecunda* (Violaceae) (Fig. S2), which was predominantly pollinated by *Ceratina japonica* (Apidae).

### Entomophily type and flower traits

**Fig. 1:**
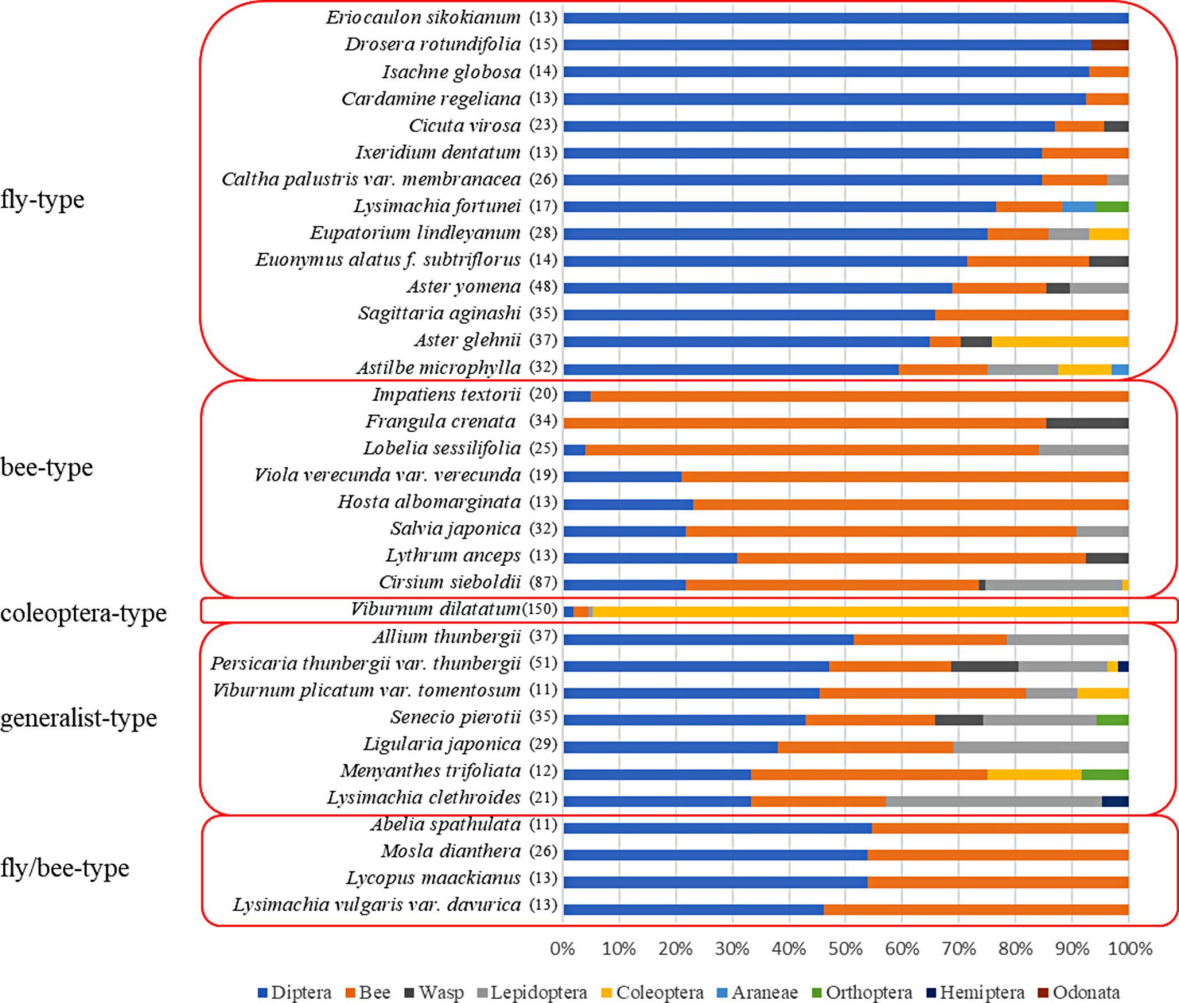
Entomophily types in Akana Wetland target plants. This figure presents the results of a hierarchical cluster analysis using Ward’s method and the Shannon-Wiener diversity index was calculated based on the order-level pollinator composition of each plant species. The plant species were classified into five entomophily types: fly-type (Diptera-dominated type), bee-type (Apoidea-dominated type), coleoptera-type (Coleoptera-dominated type), generalist-type (both flies and other insects were abundant), and fly/bee-type (Diptera-Apoidea-mixed type). The numbers in parentheses indicate the total number of captured pollinators for each plant

The 34 plant species studied were classified into 14 fly-type plants, 8 bee-type plants, 7 generalist-type plants, 4 fly/bee-type plants, and 1 coleoptera-type plant based on the cluster analysis using Ward’s method and *H’* (Fig. 1).

Open flowers were significantly more common in fly-type plants, whereas tube-shaped flowers were more common in bee-type plants (*P* < 0.001, Fisher’s exact test) (Table S7). White flowers were significantly more common in fly-type plants, whereas blue-violet flowers were more common in bee-type plants (*P* < 0.001, Fisher’s exact test) (Table S8).

### Factors affecting the flower visitation frequency of each pollinator group

**Fig. 2:**
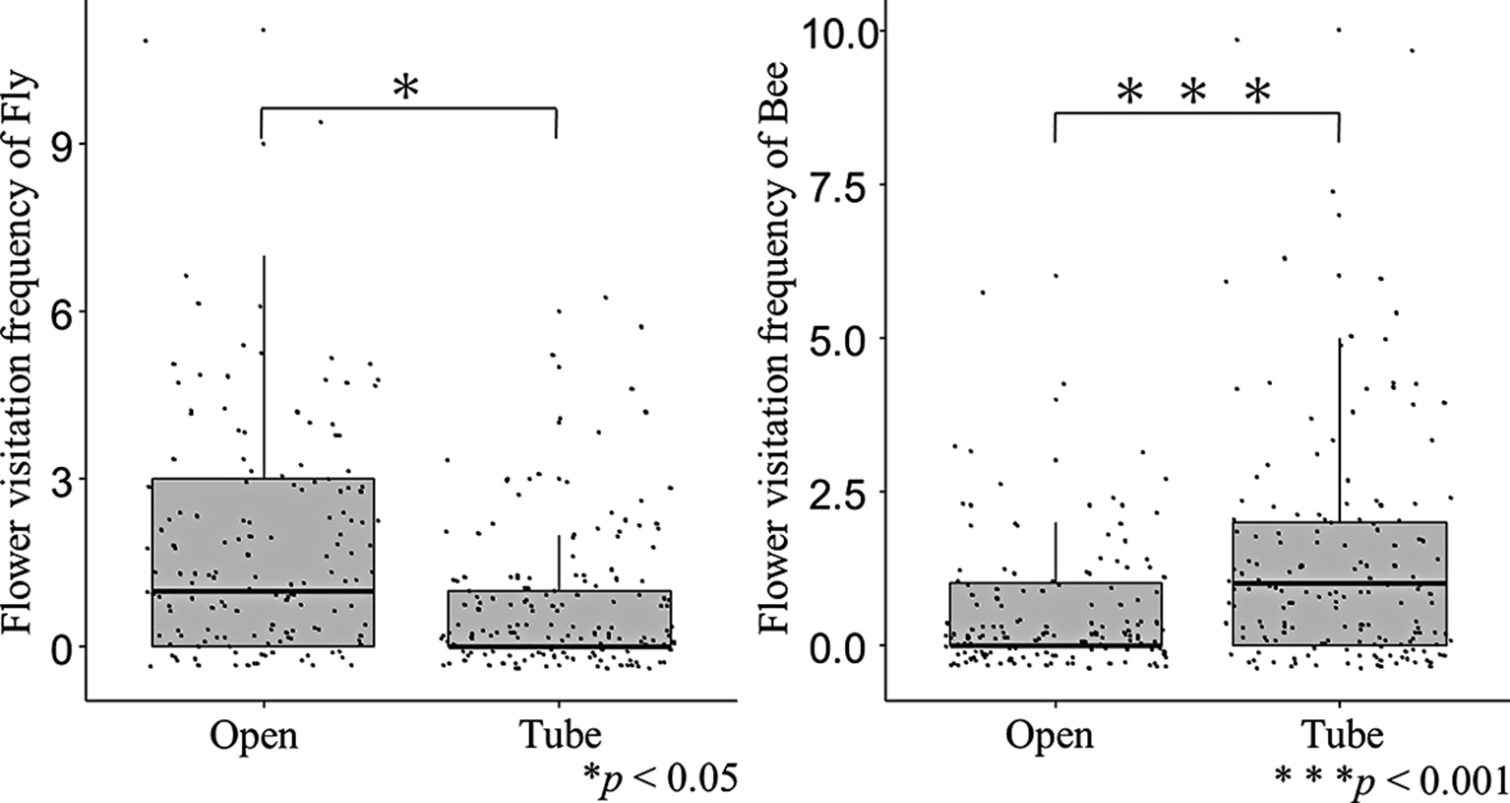
Flower morphology (open or tube-shaped) and the visitation frequency of flies and bees. We Tested for significant differences using generalized linear mixed models (GLMMs: Poisson error distribution). The details of GLMMs are shown in Table 1

**Fig. 3:**
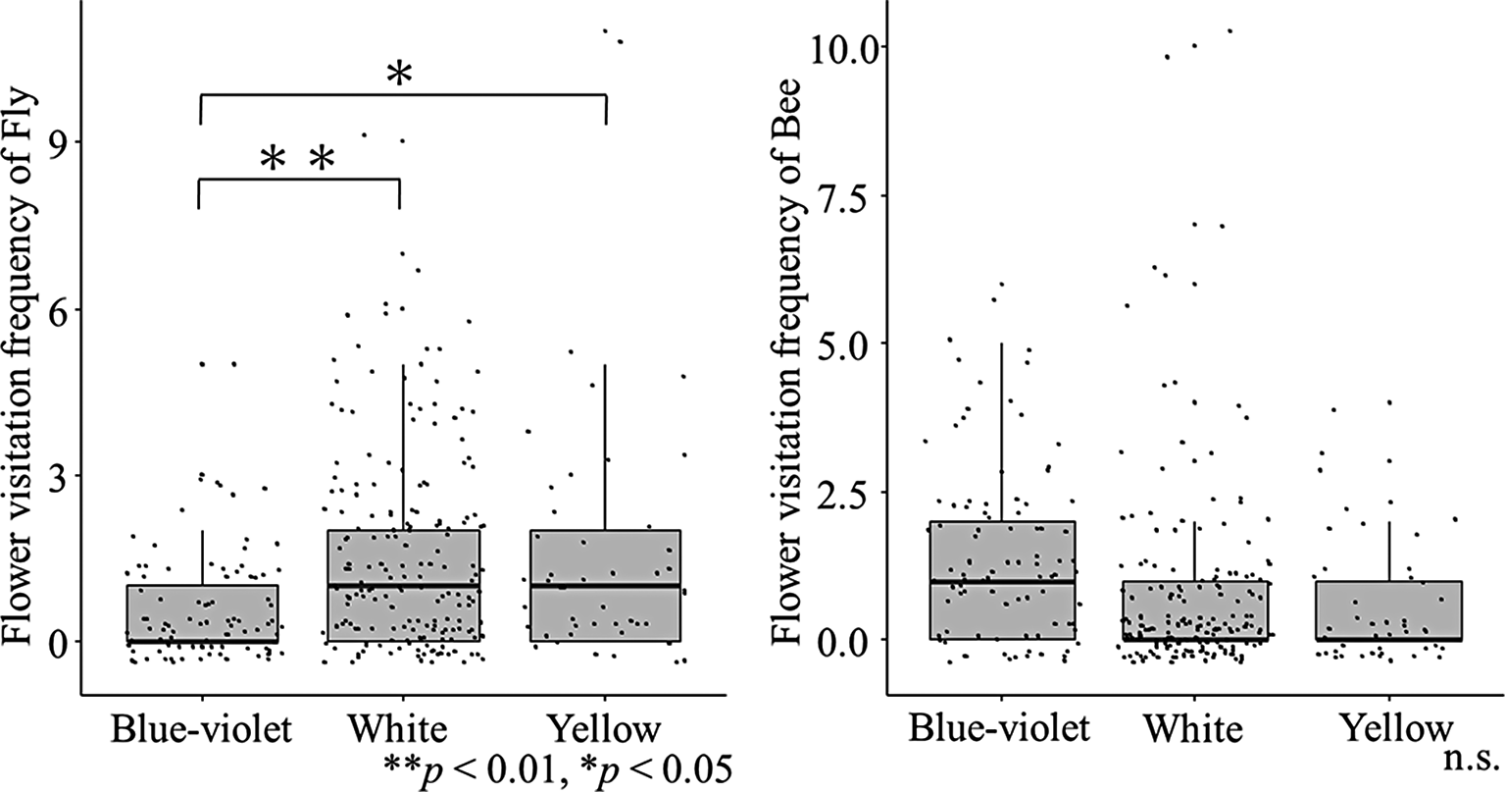
Flower color (blue-violet, white and yellow) and visitation frequency of flies and bees. We tested for significant differences using generalized linear mixed models (GLMMs) with a Poisson error distribution. More information on the GLMMs is presented in Table 1

Consistent with the findings regarding entomophily types, the results from the GLMMs also revealed that flies visited open flowers significantly more often than tube-shaped flowers (*P* < 0.05, GLMM), whereas bees showed a significant preference for tube-shaped flowers (*P* < 0.001) (Table 1; Fig. 2, GLMMs). Flies visited white (*P* < 0.01, GLMMs) and yellow (*P* < 0.01, GLMMs) flowers significantly more often than blue-violet flowers, whereas the flower visitation frequency of bees did not show significant differences across flower colors (Table 1; Fig. 3). Therefore, the prevalence of blue flowers in bee-type plants may be attributed to the lower frequency of fly visitation. Flower size had no significant effect on the flower visitation frequency of flies; however, it had a positive effect on the flower visitation frequency of bees (*P* < 0.001, GLMMs) (Table 1). Flower density exerted a positive influence on the flower visitation frequency of both flies (*P* < 0.001, GLMMs) and bees (*P* < 0.001, GLMMs).

**Table 1:** Results of the GLMM (Poisson error distribution and logarithmic link) analysis of each explanatory variable for the frequency of flies and bees visiting flowers. The random effects in the analysis included the family, genus, and species of plants, using Poisson distribution

Explanatory variable	Estimate	SE	Z value	P value
**Fly**				
Intercept	-0.90	0.65	-1.38	0.17
Shape (tube)	-0.55	0.22	-2.53	**< 0.05**
Color (white)	0.80	0.30	2.65	**< 0.01**
(yellow)	0.96	0.39	2.47	**< 0.05**
Log No. of flower	0.15	0.04	3.66	**< 0.001**
Log flower size	0.04	0.07	0.49	0.62
Habitat (forest edge)	-0.24	0.21	-1.12	0.26
Season (spring)	-0.74	0.32	-2.31	**< 0.05**
(summer)	-0.13	0.26	-0.49	0.63
Time (evening)	-0.65	0.17	-3.83	**< 0.001**
(morning)	-0.15	0.19	-0.77	0.44
**Bee**				
Intercept	-4.35	0.85	-5.12	**< 0.001**
Shape (tube)	1.42	0.31	4.60	**< 0.001**
Color (white)	0.06	0.29	0.20	0.84
(yellow)	0.60	0.38	1.59	0.11
Log No. of flower	0.23	0.06	3.77	**< 0.001**
Log flower size	0.37	0.09	3.97	**< 0.001**
Habitat (forest edge)	0.67	0.25	2.66	**< 0.01**

There was no significant difference in the flower visitation frequency of flies between plant habitat types (wetland or forest edge). However, the flower visitation frequency of bees was significantly higher for forest edge species compared to wetland species (*P* < 0.01, GLMMs) (Table 1).

With respect to the season and time of day, the flower visitation frequency of flies was significantly higher in fall than in spring (*P* < 0.05, GLMMs), and significantly higher during daytime (*P* < 0.001, GLMMs) compared to the evening.

**Fig. 4:**
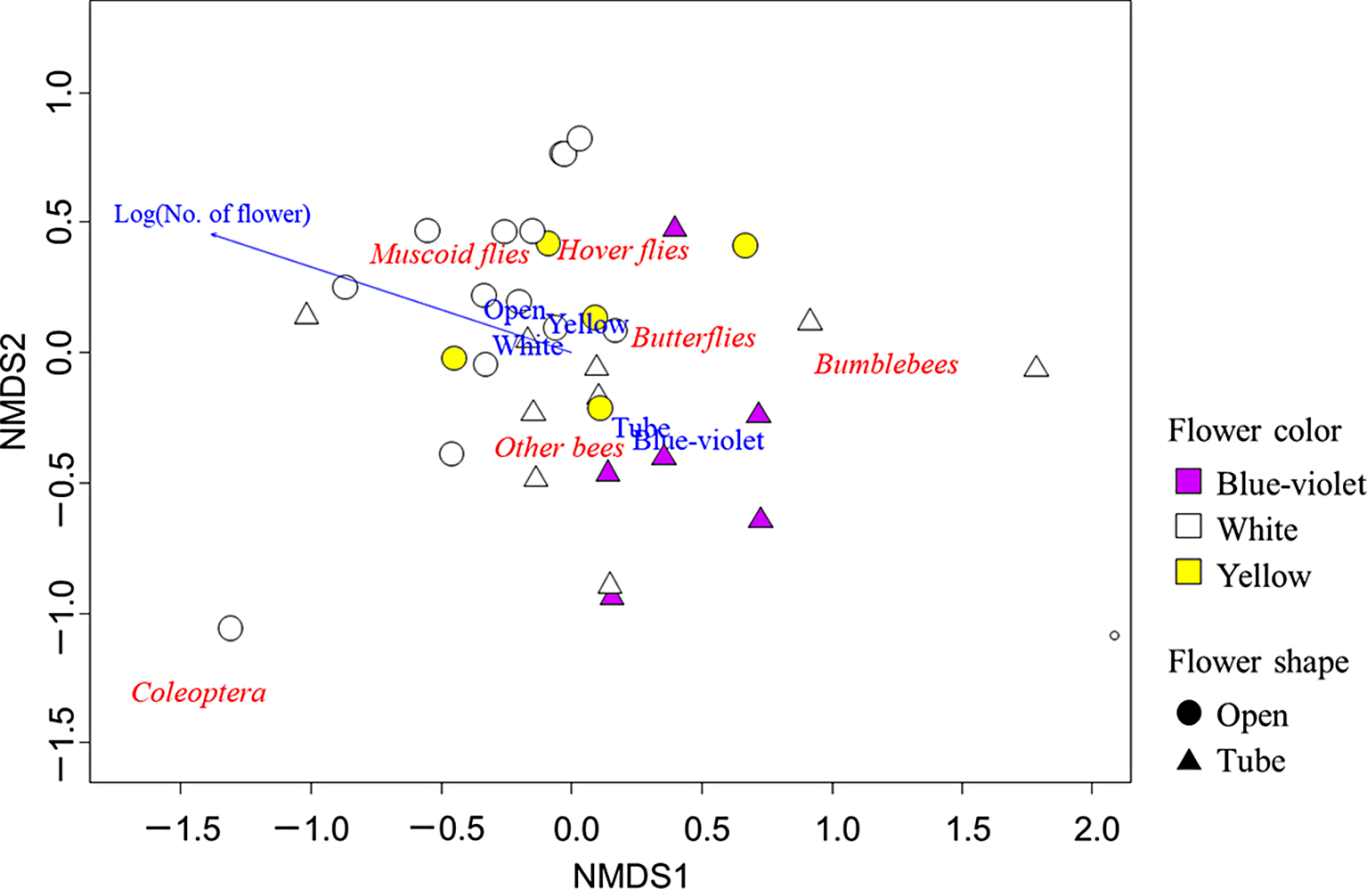
Ordination plot of non-metric multidimensional scaling (NMDS) based on the flower visitation frequency of different pollinator groups (bumblebees, other bees, muscoid flies, hover flies, coleoptera, and butterflies) for each plant species. This analysis explored the relationship between visitation frequency with flower size, density, shape (open or tube-shaped), and color (blue-violet, white/yellow). Only significant relationships from this analysis are presented. Detailed information on the NMDS is presented in Table 2

NMDS analysis showed that hover and muscoid flies exhibited a tendency to visit open, white, and yellow flowers (Fig. 4). Bumblebees and other bees showed a preference for large, tube-shaped, and blue-violet flowers (Fig. 4). Flower density (*P* < 0.001, NMDS), shape (*P* < 0.001, NMDS) and color (*P* < 0.05, NMDS) were significantly correlated with scores of two axes (Table 2).

**Table 2:** Results of the vector fitting analysis of flower size, density, shape (open or tube-shaped), and color (blue-violet, white/yellow) using non-metric multi-dimensional scaling

	NMDS1	NMDS2	*r* ^*2*^	P value
Flower size	0.99	0.12	0.14	0.11
Log (No. of flower)	-0.95	0.31	0.41	< 0.001
Flower shape	-	-	0.21	< 0.001
(Open)	-0.21	0.20	-	-
(Tube)	0.27	-0.26	-	-
Flower color	-	-	0.17	< 0.05
(Blue- violet)	0.49	-0.30	-	-
(White)	-0.17	0.06	-	-
(Yellow)	0.06	0.15	-	-

## Discussion

### Pollinator community

In the Akana Wetland, Diptera (42%) were more abundant than Hymenoptera (33%) and other pollinators in the community (Table S4). Furthermore, out of the 34 plant species analyzed, 14 were classified as fly-type plants, and 22 of the 34 species were predominantly visited by Diptera (Fig. 1). These findings support those of previous studies indicating that flies are the dominant pollinator group in wetlands, unlike forests and grasslands where bees are more prevalent (Kato and Miura 1996; Mahoro et al. 2008; Shinjo et al. 2014).

89% of the hover flies, which were the most dominant, were non-wetland-dependent species that spend their larval stage on land (Table S5). Similarly, non-wetland-dependent species were also dominant among bees, such as bumble bees, halictid bees, and other ground-nesting species. Therefore, except for *Viola verecunda* var. *verecunda*, non-wetland-dependent pollinators were dominant in all the plant species studied at the site. These findings differ from those of Kato and Miura (1996), who found that wetland pollinators are primarily insects that develop in wetlands, including aquatic and semi-aquatic hover flies that spend their larval stage in water, as well as reed-nesting *Ceratina* bees (Colletidae). Akana Wetland is a relatively small wetland (c. 2.5 ha) surrounded by secondary forests, whereas their study site was a larger paddy wetland (c. 25 ha). Therefore, many of the pollinators in their study site could be wetland species that develop in wetlands and paddy fields, whereas many of the pollinators in Akana Wetland may originate from the surrounding environment. Pindar and Raine (2023) reported that wetland are important foraging habitats for bumblebees. Stephenson et al. (2018) also reported that wetland-dependent bees were less abundant in small wetlands based on a multi-site pan trap survey. Begosh et al. (2020) reported that the visitation frequency of flies and bees in playa wetlands of Nebraska varied depending on the surrounding environment such as crop lands and natural and restored grasslands. These results suggest that wetland-dependent pollinators are not necessarily dominant in wetlands, and their dominance may depend on the wetland area and the characteristics of the surrounding environment.

We should note that our classification of larval habitat is a rough classification based on the genus level of flies and bees due to our limited knowledge on larval habitat of these species. Therefore, there would be some exceptions. For example, the majority of *Lasioglossum* are non-wetland-dependent species nesting underground, some species (e.g. *L. hartii*) are known to prefer wetland margins (Gibbs 2011). We definitely need more detailed information on their larval habitat for more accurate classification.

In contrast, the dominance of flies in wetlands, regardless of wetland or non-wetland-dependent species, is consistent with the previous studies results. This poses a question: Why are flies more prevalent in wetlands? Although Kato and Miura (1996) suggested that this could be attributed to the several fly species that inhabit wetlands during their larval stage, the explanation is likely not so simple. Another possible explanation is that wetlands have fewer flowers that attract bees. Many wetlands in Japan, including our study site, are currently small and surrounded by forests. However, it is probable that these wetlands were much larger during the Ice Age, similar to the grasslands (Ogura 2006; Ushimaru et al. 2018). In such larger wetlands, wetland-dependent flies would be dominant, rather than soil-nesting bees, as shown in the study conducted by Kato and Miura (1996). The prevalence of white and yellow flowers in the alpine zones of Japan and New Zealand, where flies are the dominant pollinators, suggests that these flower traits are adaptations to fly preferences, according to the theory of adaptation (Ishii et al. 2019). Therefore, it is reasonable to consider that flower traits that attract flies (e.g., open and white/yellow flowers) were selected in the larger wetlands during the evolutionary time scale of the ice ages and have been retained even as plants persisted in smaller wetlands, as stated by the theory of adaptation. Indeed, fly-type plants exhibit these open-shaped yellow/white flower traits (Tables S7; S8), and 29 of the 38 wetland plant species at the study site had white/yellow flowers (Table S1), which are preferred by flies.

### Relationship between the flower visitation frequency of pollinators and various flower traits

In Akana Wetland, flies visited open and white/yellow flowers, whereas bees visited tube-shaped flowers of various colors (Table 1; Figs. 2, 3 and 4). This is similar to the pattern reported in forests, grasslands, and other ecosystems (An et al. 2018; Gong and Huang 2011; Hegland and Totland 2005; Lázaro et al. 2008; McCall and Primack 1992; Shrestha et al. 2016; Westerkamp and Bockhoff 2007).

On the other hand, there were some differences between our and previous studies at the sub-group level. For example, the hover flies in our study site visited open-shaped yellow/white flowers more frequently (Fig. 4) while they tended to visit tube-shaped blue flowers in the alpine zone of Finse, Norway (Lázaro et al. 2008). Muscoid flies were dominant in Finse (Lázaro et al. 2008), the flower preference of hover flies in Finse may have changed to avoid competition with muscoid flies. Hover flies were dominant at our study site, and this is probably why they visited the open-shaped yellow/white flowers, which they originally prefer (Lunau 2014; Lunau and Maier 1995; Sutherland et al. 1999). Similarly, small solitary bees visited tube-shaped and blue-violet flowers in our study site while they visited open and white/yellow flowers more frequently in a meadow in Norway (Lázaro et al. 2008) and the Alpine Botanical Garden in China (Gong and Huang 2011). Bumblebees were more abundant than small solitary bees in the previous studies, whereas small bees were more abundant in our study (Table S4). Therefore, in our study site, small solitary bees would face less competition with bumblebees. Hiraiwa and Ushimaru (2017) also reported that short-tongued bees alter their preferences depending on the absence or presence of long-tongued bees, such as bumblebees. Therefore, realized relationships between floral traits and pollinator groups can differ depending on the composition of the dominant pollinators at each locality.

During our survey, we did not observe any pollinators on the three endangered plant species, *Platanthera nipponica* (Orchidaceae, Vulnerable in Shimane Prefecture), *Utricularia bifida* (Lentibulariaceae, Vulnerable in Shimane Prefecture), and *U. uliginosa* (Lentibulariaceae, Critically Endangered in Shimane Prefecture) (Shimane prefecture 2013). Suetsugu (2014) reported nighttime pollination by moths in the genus *Platanthera*. Because we conducted our surveys only during the daytime we may have missed them if *Platanthera* spp. rely solely on nighttime pollination. *U. praeterita* and *U. alpina* are known to rely on self-pollination without producing nectar (Chaudhary et al. 2018; Jérémie 1989). Therefore, *Utricularia bifida* and *U. uliginosa* may also rely on self-pollination.

## Conclusion

The pollinator community in the small natural wetland included in this study was characterized by the dominance of flies, which aligns with the findings of previous studies conducted in larger wetlands. However, wetland-dependent pollinators were not dominant, and over 80% of the flies and bees observed were non-wetland-dependent. This suggests that in Akana wetland, a significant portion of the pollinators may originate from the surrounding environment. Therefore, the community structure of pollinators in small wetlands might be influenced not only by the wetland itself but also by the surrounding environment. This finding has important implications considering the reduction in wetland areas caused by factors such as the abandonment of forest management, agricultural land development, urbanization, and climate change (Ferrarini et al. 2021; Fukui et al. 2012; Lui et al. 2011; Yasuda et al. 2007). However, small wetlands generally have fewer plant species than larger wetlands, and the species composition may be largely affected by local-scale topography, soil conditions, surrounding environments, and other factors (Fukui et al. 2012; Horník et al. 2012; Inoue and Mishima 2019; Watazu et al. 2022). This could be true for pollinator communities as well, which may vary from wetland to wetland. Because we investigated only one small natural wetland, it is critically important to conduct systematic surveys across many more wetlands for generalization of our conclusion. Furthermore, since our survey was limited to daytime observations, it is important to investigate pollinator activity at nighttime because some plants may rely on nocturnal pollinators. Finally, our results suggest that the dominance of flies in small wetlands would be due to the dominance of flowers preferred by flies rather than because of their larval habitats in the wetland.

### Electronic supplementary material

Below is the link to the electronic supplementary material.

Supplementary Material 1
