# Supplementary material for: Dominance of non-wetland-dependent pollinators in a plant community in a small natural wetland in Shimane, Japan
Source: J Plant Res. 2024 Jan 11;137(2):191–201. doi: 10.1007/s10265-023-01518-9 (PMC10899375; doi:10.1007/s10265-023-01518-9)
Supplement: Supplementary file 1 — Supplementary Material 1 [file 10265_2023_1518_MOESM1_ESM.doc]

**Electronic supplementary materials**

**Title:**

Dominance of non-wetland-dependent pollinators in a plant community in a small natural wetland in Shimane, Japan.

**Authors:**

Tomohiro Watazu, Masayoshi K. Hiraiwa, Masahito Inoue, Hideo Mishima, Atsushi Ushimaru, Tetsuro Hosaka

**Journal:**

Journal of Plant Research

**Corresponding author:**

Tetsuro Hosaka

(Transdisciplinary Science and Engineering Program, Graduate School of Advanced Science and Engineering, Hiroshima University, 1-5-1 Kagamiyama, Higashi-Hiroshima 7398529, Japan)

Tel: +81-82-4246929

Fax: +81-82-4246929

E-mail: hosaka3@hiroshima-u.ac.jp

**Content:**

**Tables S1-S8**

**Figs S1–S2**

**Table S1. Characteristics of flowers in the vegetation community in Akana Wetland that were included in our analyses.**

| Species | Entomophily type ^a^ | Habit ^b^ | No. of survey plot | Survey time(min) | Flower formula ^c^ | Flower size(mm^2^) | Avg No. of flower/per plot) | Flower shape ^d^ | Flower color | Red list ^e^ |
| --- | --- | --- | --- | --- | --- | --- | --- | --- | --- | --- |
| *Abelia spathulata* | FB | F | 2 | 60 | πdr | 1136 | 84 | T | White |  |
| *Allium thunbergii* | G | F | 6 | 180 | πdr | 58 | 408 | T | Blue-violet |  |
| *Aster glehnii* | F | M | 5 | 135 | πr^2^ | 148 | 919 | O | White |  |
| *Aster yomena* | F | F | 4 | 180 | πr^2^ | 1090 | 141 | O | White |  |
| *Astilbe microphylla* | F | M | 2 | 105 | πdr | 38 | 2812 | O | White |  |
| *Caltha palustris* var*. membranacea* | F | M | 6 | 285 | πr^2^ | 519 | 206 | O | Yellow | S: VU |
| *Cardamine regeliana* | F | M | 4 | 165 | lw | 19 | 531 | O | White |  |
| *Cicuta virosa* | F | M | 2 | 75 | πr^2^ | 8 | 4732 | O | White |  |
| *Cirsium sieboldii* | B | M | 26 | 480 | πdr+πr^2^ | 1710 | 15 | T | Blue-violet |  |
| *Drosera rotundifolia* | F | M | 2 | 135 | πr^2^ | 37 | 57 | O | White |  |
| *Eriocaulon sikokianum* | F | M | 5 | 120 | πr^2^ | 15 | 502 | O | White |  |
| *Euonymus alatus f. subtriflorus* | F | F | 1 | 60 | lw | 282 | 514 | O | White |  |
| *Eupatorium lindleyanum* | F | M | 5 | 135 | πdr | 7 | 6809 | T | White |  |
| *Frangula crenata* | B | F | 1 | 60 | πdr | 25 | 70 | T | White |  |
| *Hosta albomarginata* | B | M | 5 | 90 | πdr | 2655 | 27 | T | Blue-violet |  |
| *Impatiens textorii* | B | M | 7 | 120 | πdr | 1239 | 110 | T | Blue-violet |  |
| *Isachne globosa* | F | M | 2 | 105 | lw | 5 | 4266 | O | White |  |
| *Ixeridium dentatum* | F | M | 1 | 60 | πr^2^ | 225 | 319 | O | Yellow |  |
| *Ligularia japonica* | G | M | 5 | 135 | πr^2^ | 6036 | 35 | O | Yellow |  |
| *Lobelia sessilifolia* | B | M | 6 | 165 | πdr | 623 | 63 | T | Blue-violet | S: VU |
| *Lycopus maackianus* | FB | M | 9 | 165 | πdr | 14 | 341 | T | White |  |
| *Lysimachia clethroides* | G | F | 2 | 60 | πr^2^ | 73 | 166 | O | White |  |
| *Lysimachia fortunei* | F | M | 4 | 105 | πr^2^ | 61 | 89 | O | White |  |
| *Lysimachia vulgaris* var*. davurica* | FB | M | 1 | 75 | πdr | 166 | 397 | O | Yellow |  |
| *Lythrum anceps* | B | M | 2 | 135 | πr^2^ | 159 | 100 | T | Blue-violet |  |
| *Menyanthes trifoliata* | G | M | 3 | 165 | πdr | 229 | 139 | T | White | S: VU |
| *Mosla dianthera* | FB | F | 2 | 75 | πdr | 31 | 490 | T | White |  |
| *Persicaria thunbergii* var. *thunbergii* | G | M | 16 | 300 | πdr | 33 | 239 | T | White |  |
| *Platanthera nipponica* var. *nipponica* |  | M | 2 | 105 | hw | 29 | 25 | T | Yellow | S: CR+EN |
| *Pogonia japonica* |  | M | 1 | 105 | πdr+ lw/2 | 365 | 10 | T | Blue-violet | MOE: NT, S: CR+EN |
| *Sagittaria aginashi* | F | M | 5 | 135 | lw/2 | 352 | 9 | O | White | MOE: NT, S: CR+EN |
| *Salvia japonica* | B | F | 7 | 195 | πdr+lw | 105 | 120 | T | Blue-violet |  |
| *Senecio pierotii* | G | M | 4 | 90 | πr^2^ | 598 | 81 | O | Yellow |  |
| *Utricularia bifida* |  | M | 1 | 60 | πdr+lw | 58 | 22 | T | Yellow | S: VU |
| *Utricularia uliginosa* |  | M | 1 | 60 | πdr+lw | 24 | 33 | T | Blue-violet | MOE: NT, S: CR+EN |
| *Viburnum dilatatum* | C | F | 1 | 75 | πr^2^ | 31 | 2492 | O | White |  |
| *Viburnum plicatum* var. *tomentosum* | G | F | 1 | 120 | lw | 475 | 344 | O | White |  |
| *Viola verecunda* var*. verecunda* | B | M | 7 | 195 | πdr | 187 | 114 | T | White |  |

^a^ F, fly-type; B, bee-type; FB, fly/bee-type, G, generalist-type; C, coleoptera-type

^b^ M, Marsh; F, Forest edge

^c^ πr^2^ (r = radius) circular flower, lw (l = length, w = width), square flower; lw/2, triangular flower; πdr, πdr + πr^2^, πdr + lw, πdr + lw/2 (d = radius of fan shape with cone expanded), conical flower

^d^ O, open; T, tube-shaped

^e^ MOE, Ministry of the Environment red list (2020); S, Shimane Prefecture red list (2013)

**Table S2. Frequency of visitation of pollinators, entomophily type, and diversity index for the target plants.**

| Species | Entomophily type ^a^ | Diversity index ^b^ | No. of insects | All insects  (per 15min) | Fly  (per 15min) | Bee  (per 15min) | All insects (per 100 cm^2^  flower area ^c^ per 15min) | Fly  (per 100 cm^2^ flower area ^c^ per 15min) | Bee  (per 100 cm^2^ flower area ^c^ per 15min) |
| --- | --- | --- | --- | --- | --- | --- | --- | --- | --- |
| *Abelia spathulata* | FB | 0.99 | 11 | 2.75 | 1.5 | 1.25 | 0.29 | 0.16 | 0.13 |
| *Allium thunbergii* | G | 1.48 | 37 | 3.08 | 1.58 | 0.83 | 1.31 | 0.67 | 0.35 |
| *Aster glehnii* | F | 1.36 | 37 | 4.11 | 2.67 | 0.22 | 0.30 | 0.20 | 0.02 |
| *Aster yomena* | F | 1.33 | 48 | 4 | 2.75 | 0.67 | 0.26 | 0.18 | 0.04 |
| *Astilbe microphylla* | F | 1.72 | 32 | 4.57 | 2.71 | 0.71 | 0.42 | 0.25 | 0.07 |
| *Caltha palustris* var*. membranacea* | F | 0.74 | 26 | 1.37 | 1.16 | 0.16 | 0.13 | 0.11 | 0.01 |
| *Cardamine regeliana* | F | 0.39 | 13 | 1.18 | 1.09 | 0.09 | 1.20 | 1.11 | 0.09 |
| *Cicuta virosa* | F | 0.68 | 23 | 4.6 | 4 | 0.4 | 1.16 | 1.01 | 0.10 |
| *Cirsium sieboldii* | B | 1.61 | 87 | 2.72 | 0.59 | 1.41 | 1.07 | 0.23 | 0.55 |
| *Drosera rotundifolia* | F | 0.35 | 15 | 1.67 | 1.56 | 0 | 8.02 | 7.49 | 0.00 |
| *Eriocaulon sikokianum* | F | 0 | 13 | 1.63 | 1.63 | 0 | 2.17 | 2.17 | 0.00 |
| *Euonymus alatus f. subtriflorus* | F | 1.09 | 14 | 3.5 | 2.5 | 0.75 | 0.24 | 0.17 | 0.05 |
| *Eupatorium lindleyanum* | F | 1.2 | 28 | 3.11 | 2.33 | 0.33 | 0.68 | 0.51 | 0.07 |
| *Frangula crenata* | B | 0.6 | 34 | 8.5 | 0 | 7.25 | 48.67 | 0.00 | 41.52 |
| *Hosta albomarginata* | B | 0.78 | 13 | 2.17 | 0.5 | 1.67 | 0.30 | 0.07 | 0.23 |
| *Impatiens textorii* | B | 0.29 | 20 | 2.5 | 0.13 | 2.38 | 0.18 | 0.01 | 0.17 |
| *Isachne globosa* | F | 0.37 | 14 | 2 | 1.86 | 0.14 | 0.98 | 0.91 | 0.07 |
| *Ixeridium dentatum* | F | 0.62 | 13 | 3.25 | 2.75 | 0.5 | 0.45 | 0.38 | 0.07 |
| *Ligularia japonica* | G | 1.58 | 29 | 3.22 | 1.22 | 1 | 0.15 | 0.06 | 0.05 |
| *Lobelia sessilifolia* | B | 0.87 | 25 | 2.27 | 0.09 | 1.82 | 0.58 | 0.02 | 0.46 |
| *Lycopus maackianus* | FB | 1 | 13 | 1.18 | 0.64 | 0.55 | 2.52 | 1.36 | 1.16 |
| *Lysimachia clethroides* | G | 1.76 | 21 | 5.25 | 1.75 | 1.25 | 4.36 | 1.45 | 1.04 |
| *Lysimachia fortunei* | F | 1.14 | 17 | 2.43 | 1.86 | 0.29 | 4.51 | 3.45 | 0.53 |
| *Lysimachia vulgaris* var*. davurica* | FB | 1 | 13 | 2.6 | 1.2 | 1.4 | 0.39 | 0.18 | 0.21 |
| *Lythrum anceps* | B | 1.24 | 13 | 1.44 | 0.44 | 0.89 | 0.91 | 0.28 | 0.56 |
| *Menyanthes trifoliata* | G | 1.78 | 12 | 1.09 | 0.36 | 0.45 | 0.34 | 0.11 | 0.14 |
| *Mosla dianthera* | FB | 1 | 26 | 5.2 | 2.8 | 2.4 | 3.46 | 1.86 | 1.60 |
| *Persicaria thunbergii* var. *thunbergia* | G | 1.99 | 51 | 2.55 | 1.2 | 0.55 | 3.20 | 1.51 | 0.69 |
| *Platanthera nipponica* var*. nipponica* |  |  | 0 | 0 | 0 | 0 | 0.00 | 0.00 | 0.00 |
| *Pogonia japonica* |  |  | 3 | 0.43 | 0.29 | 0.14 | 1.16 | 0.77 | 0.39 |
| *Sagittaria aginashi* | F | 0.93 | 35 | 3.89 | 2.67 | 1.22 | 11.99 | 8.22 | 3.77 |
| *Salvia japonica* | B | 1.17 | 32 | 2.46 | 0.54 | 1.69 | 1.96 | 0.43 | 1.34 |
| *Senecio pierotii* | G | 2.01 | 35 | 5.83 | 2.5 | 1.33 | 1.21 | 0.52 | 0.28 |
| *Utricularia bifida* |  |  | 0 | 0 | 0 | 0 | 0.00 | 0.00 | 0.00 |
| *Utricularia uliginosa* |  |  | 0 | 0 | 0 | 0 | 0.00 | 0.00 | 0.00 |
| *Viburnum dilatatum* | C | 0.38 | 150 | 30 | 0.6 | 0.8 | 3.89 | 0.08 | 0.10 |
| *Viburnum plicatum* var*. tomentosum* | G | 1.68 | 11 | 1.38 | 0.63 | 0.5 | 0.08 | 0.04 | 0.03 |
| *Viola verecunda* var*. verecunda* | B | 0.74 | 19 | 1.46 | 0.31 | 1.15 | 0.69 | 0.14 | 0.54 |

^a^ F, fly-type; B, bee-type; FB, fly/bee-type, G, generalist-type; C, coleoptera-type

^b^ The Shannon-Wiener diversity index was used at the order level of pollinators.

^c^ The flower area is the area of a single flower multiplied by the number of flowers in the plot.

**Table S3. Comparison of AIC value for each model with different explanatory variables in the GLMMs.**

| Response variable | Explonatary variables | | | | | | | AIC |
| --- | --- | --- | --- | --- | --- | --- | --- | --- |
|  | 1 | 2 | 3 | 4 | 5 | 6 | 7 |  |
| The flower visitation frequency of flies | Shape | Clolor | Log No. of flower | Log flower size | Habitat | Seson | Time | 979.9 |
|  | Shape | Clolor | Log No. of flower | Log flower size | Habitat | Time |  | 981.6 |
|  | Shape | Clolor | Log No. of flower | Log flower size | Habitat | Seson |  | 992.6 |
|  | Shape | Clolor | Log No. of flower | Log flower size | Habitat |  |  | 993.8 |
| The flower visitation frequency of bees | Shape | Clolor | Log No. of flower | Log flower size | Habitat |  |  | 829.2 |
|  | Shape | Clolor | Log No. of flower | Log flower size | Habitat | Time |  | 829.2 |
|  | Shape | Clolor | Log No. of flower | Log flower size | Habitat | Seson |  | 832.6 |
|  | Shape | Clolor | Log No. of flower | Log flower size | Habitat | Seson | Time | 832.7 |

**Table S4. Ratio of pollinator groups classified referring to Lázaro et al. (2008).**

| Pollinator | | individual | rate |
| --- | --- | --- | --- |
| Diptera | Hover flies | 268 | 27.3 |
|  | Muscoid flies | 105 | 10.7 |
|  | Other flies | 28 | 2.8 |
|  | Beeflies | 9 | 0.9 |
| Hymenoptera | Other bees | 178 | 18.1 |
|  | Bumblebees | 111 | 11.3 |
|  | Wasps | 23 | 2.3 |
|  | Honey bees | 9 | 0.9 |
| Coleoptera | Coleoptera | 161 | 16.4 |
| Butterfly | Butterflies | 82 | 8.3 |
| Other | Orthoptera | 4 | 0.4 |
|  | Araneae | 2 | 0.2 |
|  | Hemipterans | 2 | 0.2 |
|  | Odonata | 1 | 0.1 |
| Total |  | 983 | 100.0 |

**Table S5. A list of hover fly tribes identified at Akana Wetland, including their larval habitat and relative abundance.**

| Tribe | Larval habitat ^a^ | No. of species | Individual |
| --- | --- | --- | --- |
| Syrphini | T | 8 | 168 |
| Paragini | T | 1 | 47 |
| Eristalini | A | 7 | 29 |
| Cheilosiini | T | 3 | 11 |
| Eumerini | T | 2 | 6 |
| Melanostomatini | T | 2 | 5 |
| Milesiini | T | 2 | 2 |
| Total |  | 25 | 268 |

^a^ T, Terrestrial; A, Aquatic

**Table S6. A list of bee genera identified at Akana Wetland, including their nest site, habitat, and relative abundance.**

| Family | Genus | Sub-taxonomic groups | Nest site ^a^ | Habitat ^b^ | No. of species | Individual |
| --- | --- | --- | --- | --- | --- | --- |
| Apidae | *Bombus* | Bumblebees | g | n-w | 4 | 111 |
|  | *Amegilla* | Other bees | g | n-w | 1 | 17 |
|  | *Apis* | Honey bees | h | n-w | 1 | 1 |
| Anthophoridae | *Ceratina* | Other bees | s | w | 3 | 48 |
|  | *Xylocopa* | Other bees | w | n-w | 1 | 17 |
|  | *Nomada* | Other bees | p | n-w | 4 | 4 |
| Halictidae | *Lasioglossum* | Other bees | g | n-w | 8 | 48 |
|  | *Halictus* | Other bees | g | n-w | 2 | 13 |
| Megechilidae | *Megachile* | Other bees | g, s | n-w | 1 | 21 |
|  | *Coelioxys* | Other bees | p | n-w | 1 | 2 |
| Melittidae | *Macropis* | Other bees | g | n-w | 1 | 6 |
|  | *Melitta* | Other bees | g | n-w | 1 | 3 |
| Colletidae | *Hylaeus* | Other bees | s | w | 1 | 4 |
| Andrenidae | *Andrena* | Other bees | g | n-w | 2 | 2 |
| Total |  |  |  |  | 31 | 297 |

^a^ g, underground; h, tree hollows; p, cleptoparasitic, s, stem hollows; w, tree burrows bored by the bee

^b^ w, wetland-dependent; n-w, non-wetland-dependent

**Table S7. Number of species with specific flower shapes (open or tube-shaped) in the entomophily type classified in Fig. 1. The numbers in parentheses indicate the number of plant species of each entomophily type.**

| Entomophily type | Open | Tube |
| --- | --- | --- |
| fly-type (14) | 13 | 1 |
| bee-type (8) | 0 | 8 |
| generalist-type (7) | 4 | 3 |
| fly/bee-type (4) | 1 | 3 |

Results of Fisher’s exact probability test for flower shape proportions of each entomophily type.

fly-type and bee-type (P < 0.001)

　fly-type and generalist-type (P = 0.09)

fly-type and fly/bee-type (P < 0.05)

bee-type and generalist-type (P < 0.05)

bee-type and fly/bee-type (P = 0.33)

generalist-type and fly/bee-type (P = 0.55)

**Table S8. Number of species of the flower color (blue-violet, white/yellow) in the entomophily type classified in Fig. 1. The numbers in parentheses indicate the number of plant species of each entomophily type.**

| Entomophily type | Blue-violet | White | Yellow |
| --- | --- | --- | --- |
| fly-type (14) | 0 | 12 | 2 |
| bee-type (8) | 6 | 2 | 0 |
| generalist-type (7) | 1 | 4 | 2 |
| fly/bee-type (4) | 0 | 3 | 1 |

Results of Fisher’s exact probability test for flower color proportions of each entomophily type.

fly-type and bee-type (P < 0.001)

fly-type and generalist-type (P = 0.25)

fly-type and fly/bee-type (P = 1)

bee-type and generalist-type (P < 0.05)

bee-type and fly/bee-type (P < 0.05)

generalist-type and fly/bee-type (P = 1)

**Fig. S1. Results of the hierarchical cluster analysis using Ward’s method based on the order-level pollinator composition of each plant species.**

**
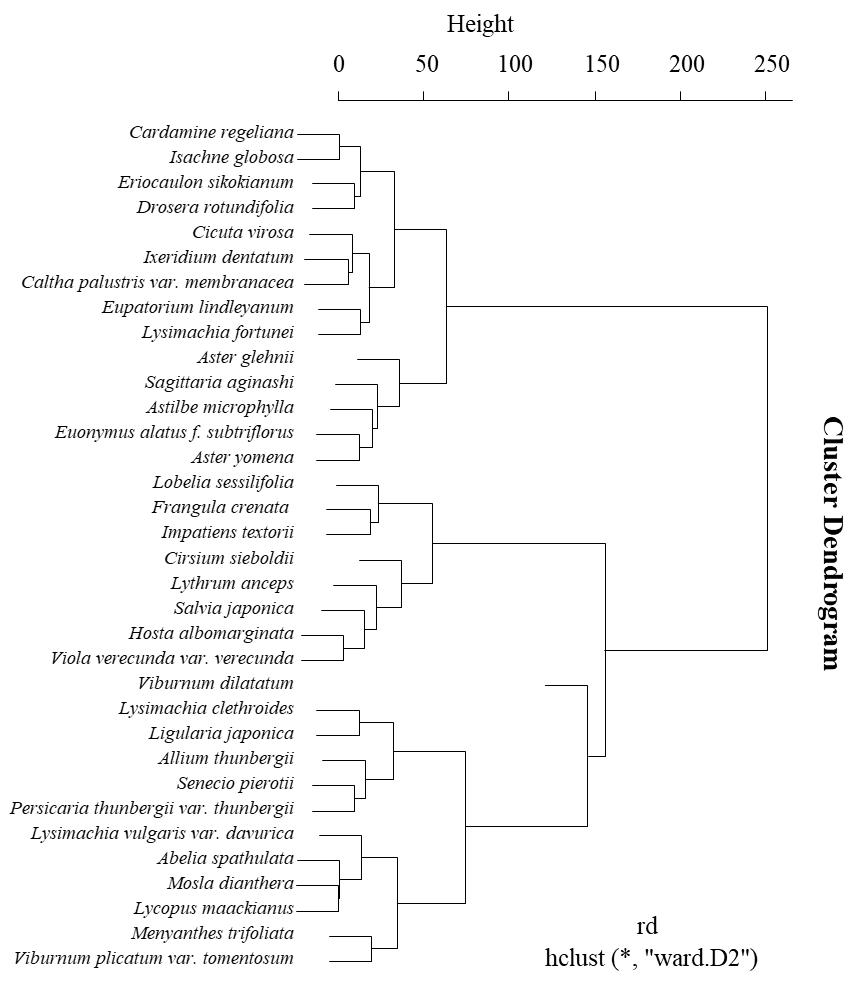
**


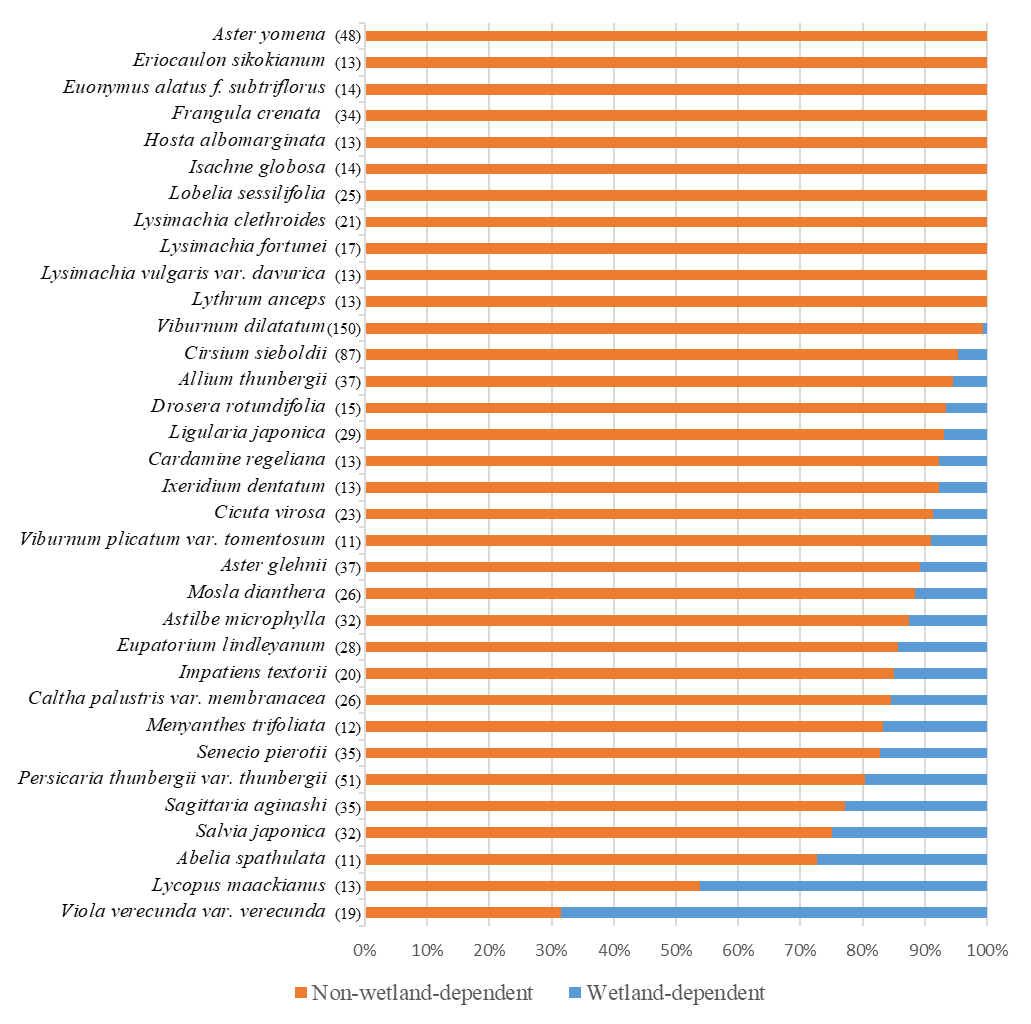
**Fig. S2. Percentage of habitats (wetland or non-wetland-dependent, including forest and grassland) at the larval stage for pollinators of 34 plant species in Akana Wetland. Numbers in parentheses indicate the total number of captured pollinators for each plant.**

**References**

Ministry of the Environment of Japan (2020) Red List of threatened wildlife of Japan, Vascular Plants, revised edition (in Japanese). Ministry of the Environment of Japan. https://ikilog.biodic.go.jp/Rdb/booklist. Accessed 6 January 2023

Shimane prefecture (2013) Red list of threatened wildlife of Shimane, vascular plants, revised edition (in Japanese). Shimane prefecture. https://www.pref.shimane.lg.jp/infra/nature/shizen/yasei/red-data/. Accessed 6 January 2023
